# Supplementary material for: When Stroke Strikes Early: Unusual Causes of Intracerebral Hemorrhage in Young Adults
Source: J Clin Med. 2025 Nov 29;14(23):8475. doi: 10.3390/jcm14238475 (PMC12693425; doi:10.3390/jcm14238475)
Supplement: Supplementary file 1 [file jcm-14-08475-s001.zip › jcm-3924351-supplementary.pdf]

Journal of Clinical Medicine
MDPI

Supplementary Material:

Table S1. ICD-10-CM Code Definitions for Rare Etiologies of Intracerebral Hemorrhage

Etiology	ICD-10-CM Codes	Description / Notes
Arteriovenous malformation / Aneurysm	Q28.2, Q28.3, I67.1, I72.x	Congenital or acquired AVM, cerebral or intracranial aneurysm
Brain tumor	C70–C72, C79.3, D33.x	Primary or secondary malignant or benign brain neoplasm
Moyamoya disease	I67.5	Stenosis/occlusion of circle of Willis arteries
Sickle cell disease	D57.x	Includes Hb-SS, Hb-SC, and other variants
Infection	G00–G03	Meningitis, encephalitis, brain abscess with hemorrhagic potential
Vasculitis	I77.6, M30–M31	Isolated CNS vasculitis or systemic vasculitic syndromes
Cerebral venous sinus thrombosis (CVT)	I67.6, G08	Intracranial venous sinus or cortical vein thrombosis
Pregnancy-related ICH	O10–O16, O22.5, O87.3	Hypertensive disorders of pregnancy, pregnancy-associated CVT
Exclusions	S06.x, S02.x	Traumatic brain injury or skull fracture codes excluded

Note: Etiology flags were not mutually exclusive; multiple rare etiologies could be present for a single hospitalization.

Supplementary Table S2. Discharge Disposition by Age Group

Measure	18-39 years	<45 years	<50 years	≥40 years	p-value (18-39 vs ≥40)
Weighted N (%)	4,012 (5.3%)	5,686 (7.4%)	7,982 (10.5%)	72,252 (94.7%)	–
Mean age (± SD)	28.9 ± 6.8	33.4 ± 7.8	36.7 ± 8.4	69.2 ± 11.3	<0.001
In-hospital mortality (%)	**15.7**	**16.7**	**17.5**	21.7	<0.001
Mean length of stay (days)	12.1 ± 0.4	11.8 ± 0.4	11.6 ± 0.3	8.7 ± 0.2	<0.001
Mean hospital charges (USD)	228,000 ± 8,500	221,000 ± 7,900	214,000 ± 7,600	125,000 ± 4,200	<0.001
Discharge home (%)	42.1	39.6	38.2	17.2	<0.001
Transfer to rehabilitation facility (%)	24.7	26.1	27.3	34.8	<0.001
Transfer to skilled nursing facility (%)	4.2	5.1	5.8	11.7	<0.001
Home health care (%)	5.8	6.0	6.3	10.7	<0.001
Left against medical advice (%)	2.0	1.8	1.7	1.0	0.02
Other / unknown (%)	5.5	5.3	5.1	2.9	0.01

Percentages are weighted using NIS discharge weights (2016–2022). “Other” includes hospice and non-specified transfers.

Supplementary Table S3. Multivariable Logistic Regression for In-Hospital Mortality (Weighted Model)

Variable	Adjusted Odds Ratio (aOR)	95% Confidence Interval	p-value
Age ≥ 40 years	1.67	1.52–1.84	<0.001
Coagulopathy	1.34	1.27–1.42	<0.001
Chronic kidney disease	1.14	1.09–1.20	<0.001
Coronary artery disease	1.28	1.19–1.38	<0.001
Heart failure	1.21	1.12–1.30	<0.001
Atrial fibrillation	1.18	1.09–1.28	<0.001
Female sex	1.01	0.96–1.06	0.68
Race (non-White)	0.95	0.88–1.02	0.12
Hypertension	0.98	0.93–1.03	0.32
Diabetes mellitus (E08–E13)	0.99	0.93–1.05	0.61
Obesity	0.92	0.84–1.01	0.08
Hyperlipidemia	0.89	0.82–0.96	0.003
Alcohol use	0.97	0.88–1.06	0.41
Substance use	0.94	0.88–1.01	0.09
